# Tools for retargeting proteins within *Aspergillus nidulans*

**DOI:** 10.1371/journal.pone.0189077

**Published:** 2017-12-01

**Authors:** Subbulakshmi Suresh, Leymaan Abdurehman, Aysha H. Osmani, Stephen A. Osmani

**Affiliations:** 1 Department of Molecular Genetics, The Ohio State University, Columbus, Ohio, United States of America; 2 Laboratory of Chemistry and Cell Biology, The Rockefeller University, New York, New York, United States of America; Woosuk University, REPUBLIC OF KOREA

## Abstract

Endogenously tagging proteins with green fluorescent protein (GFP) enables the visualization of the tagged protein using live cell microscopy. GFP-tagging is widely utilized to study biological processes in model experimental organisms including filamentous fungi such as *Aspergillus nidulans*. Many strains of *A*. *nidulans* have therefore been generated with different proteins endogenously tagged with GFP. To further enhance experimental approaches based upon GFP-tagging, we have adapted the GFP Binding Protein (GBP) system for *A*. *nidulans*. GBP is a genetically encoded Llama single chain antibody against GFP which binds GFP with high affinity. Using gene replacement approaches, it is therefore possible to link GBP to anchor proteins, which will then retarget GFP-tagged proteins away from their normal location to the location of the anchor-GBP protein. To facilitate this approach in *A*. *nidulans*, we made four base plasmid cassettes that can be used to generate gene replacement GBP-tagging constructs by utilizing fusion PCR. Using these base cassettes, fusion PCR, and gene targeting approaches, we generated strains with SPA10-GBP and Tom20-GBP gene replacements. These strains enabled test targeting of GFP-tagged proteins to septa or to the surface of mitochondria respectively. SPA10-GBP is shown to effectively target GFP-tagged proteins to both forming and mature septa. Tom20-GBP has a higher capacity to retarget GFP-tagged proteins being able to relocate all Nup49-GFP from its location within nuclear pore complexes (NPCs) to the cytoplasm in association with mitochondria. Notably, removal of Nup49-GFP from NPCs causes cold sensitivity as does deletion of the *nup49* gene. The cassette constructs described facilitate experimental approaches to generate precise protein-protein linkages in fungi. The *A*. *nidulans* SPA10-GBP and Tom20-GBP strains can be utilized to modulate other GFP-tagged proteins of interest.

## Introduction

Having the ability to experimentally link proteins to each other within cells provides several experimental avenues to interrogate protein function. For example, it enables experiments to test the effects of preventing proteins from locating to their normal subcellular locations by artificially re-targeting them to another cellular location. Additionally, if the linked target proteins locate stably to specific structures it is possible to experimentally bridge the structures to which the two proteins belong. For example, using this approach we recently provided linkage between nuclear pore complexes (NPCs) and nuclear chromatin in the model fungus *Aspergillus nidulans* [1]. This was done to test the idea that the Nup2, a NPC protein which uniquely transitions from NPCs to chromatin during mitosis, helps mitotic segregation of NPC by naturally linking mitotic chromatin to NPCs. By providing an artificial chromatin-NPC bridge we were able to show that NPCs were segregated to daughter nuclei in the absence of Nup2, although without this link NPCs are mis-segregated into the cytoplasm after mitosis [1]. To generate the chromatin-NPC bridge we used the GFP Binding Protein (GBP) system [2]. The GFP-GBP system has been developed for use in human cells and has also been successfully employed in plants, fission yeast and prokaryotes but has had limited application in filamentous fungi [3–6]. This system is based on a genetically encoded Llama single chain antibody against GFP [2, 3]. GBP is a 13-kDa protein, almost half the size of GFP itself, which binds GFP with high affinity and can be expressed as a fusion protein to target proteins of interest. GFP and GBP bind in a stoichiometric manner with one molecule of GBP binding to one molecule of GFP in a stable complex [2]. The dissociation constant (Kd) lies within the picomolar range which is comparable to affinities in conventional antigen-antibody interactions [3]. GBP recognizes GFP and YFP but not CFP or DsRed derivatives, such as mCherry or mRFP [2].

In the current work, we report the generation of strains (Table 1, Strains used in this study) with GBP protein fusions to enable retargeting of GFP-tagged proteins to either septa or mitochondria. Validating the usefulness of this approach, we report that retargeting the NPC protein Nup49-GFP to the mitochondria via Tom20 mimics the loss of Nup49 function as seen by marked cold-sensitivity [7]. To enable generation of additional retargeting GBP constructs via fusion PCR [8] we also constructed plasmids containing the GBP coding sequence upstream of either the *pyrG* or the *pyroA* selective nutritional transformation markers to select for *Aspergillus* transformants. One set of cassettes encodes mRFP linked to the GBP sequence to enable visualization of the generated target protein-mRFP-GBP chimeras (Table 2, Plasmids used in this study). These cassettes facilitate the generation of GBP gene replacement tagging constructs using fusion PCR to integrate GBP at the C-termini of chosen retargeting proteins at their endogenous locus. In our previously generated cassettes for C-terminal tagging, we incorporated sequences to encode a glycine-alanine linker (GA_5_ linker) [9]. We further included a common sequence at the cassettes 3’ ends [10]. These common sequences have been incorporated in the four new GBP cassettes such that the same set of primers that have previously been used for fluorescent or affinity purification protein tagging can be employed [9, 11, 12]. The resources facilitate experiments based upon experimentally linking proteins to each other within *Aspergillus* and other fungal cells.

**Table 1:** Strains used in this study.

Strain	Genotype	Reference
SGS134	*SPA10-GBP-mRFP*::*pyroA*^*Af*^ *(pyroA4); pyrG89; wA3; fwA1; chaA1; Δmus51*::*argB (argB2); SE15; nirA14*	Transformant of SO451
SGS136	*(p)gpdA*::*NLS-GFP*::*pyrG*^*Af*^ *(pyrG89); SPA10-GBP*::*pyroA*^*Af*^ *(pyroA4); sE15; nirA14; ΔnKuA*::*argB (argB2); fwA1; chaA1; wA3*	Transformant of KF430
SGS141	*(p)AN1553*::*GFP-S-tag*::*pyrG*^*Af*^*; SPA10-GBP*::*pyroA*^*Af*^ *(pyroA4); ΔnKuA*::*argB; (argB2); ΔyA*::*NLS-DsRed; nirA14?; sE15?*	Transformant of KF491
SGS365	*Tom20-GBP-mRFP*::*pyroA*^*Af*^ *(pyroA4); ΔyA*::*NLS-DsRed; pyrG89; Δmus51*:: *argB (argB2); wA2/wA3; fwA1?; chaA1?; sE15*	Transformant of SGS197
SGS367	*Nup49-GFP-pyrG*^*Af*^ *(pyrG89); ΔyA*::*NLS-DsRed; pyroA4; Δmus51*:: *argB (argB2); wA2/wA3; fwA1?; chaA1?; sE15*	Transformant of SGS197
SGS374	*Tom20-GBP-mRFP*::*pyroA*^*Af*^ *(pyroA4); Nup49-GFP*::*pyrG*^*Af*^ *(pyrG89); ΔyA*::*NLS-DsRed; Δmus51*:: *argB (argB2); wA2/wA3; fwA1?; chaA1?; sE15*	Transformant of SGS365
KF430	*(p)gpdA*::NLS-GFP::*pyr4; (pyrG89); pyroA4; sE15; nirA14;* *ΔnKuA*::*argB; (argB2); fwA1; chaA1; wA3*	[13]
KF491	*(p)AN1553*::GFP-S-tag::*pyrG*^*Af*^*; ΔnKuA*::*argB; (argB2); pyroA4;ΔyA*::*NLS-DsRed; nirA14?; sE15?*	[13]
CDS746	*wA3; yA2?*	[1]
CDS1008	*ΔnKuA*^*Ku70*^::*argB; pyroA4; argB2; sE15; nirA14; wA3; fwA1; chaA1*	[14]
SO759	*yA2; ΔAn-nup49*::*pyrG*^*Af*^	[7]
SO1749	*An-nup49-GFP*::*pyrG*^*Af*^ *(pyrG89); SPA10-GBP-mRFP*::*pyroA*^*Af*^ *(pyroA4); wA3;*	SO594 x SGS134
SO1752	*tom20-GBP-mRFP*::*pyroA*^*Af*^ *(pyroA4); pyrG89; se15; Δ ku70*::*argB (argB2); wA3 or wA2*.	S0594 x SGS365
SO1753	*An-nup49-GFP*::*pyrG*^*Af*^ *(pyrG89); argB2; sE15;pyroA4? wA3/wA2*.	S0594 x SGS365
SO451	*pyrG89; wA3; argB2; ΔnkuA*::*argB pyroA4; se15 nirA14 chaA1 fwa1*	[7]
SO594	*pyrG89; wA3; argB2; nup49-GFP*::*pyrG*^*Af*^*; fwA1; nirA14*	[7]
SGS197	*ΔyA*::*NLS-DsRed; pyrG89; pyroA4; Δmus51*:: *argB (argB2); wA2/wA3; fwA1?; chaA1?; sE15*	[1]

Some nutritional and color markers could be covered by, or be recessive to, other markers in the strain and are designated by a question mark.

Af represents genes from *A*. *fumigatus* used for complementation of the corresponding *A*. *nidulans* nutritional mutations.

x Indicates a genetic cross between the mentioned strains.

**Table 2:** Plasmids used in this study.

Plasmid	Construct	Reference
pFNO3	GFP::*pyrG*^Af^	[9]
pGS1	GBP::*pyrG*^Af^	This study
pGS2	GBP::*pyroA*^Af^	This study
pGS3	GBP-mRFP::*pyrG*^Af^	This study
pGS4	GBP-mRFP::*pyroA*^Af^	This study

## Results and discussion

### Design of the GBP cassette plasmid vectors for protein retargeting

**Fig 1:**
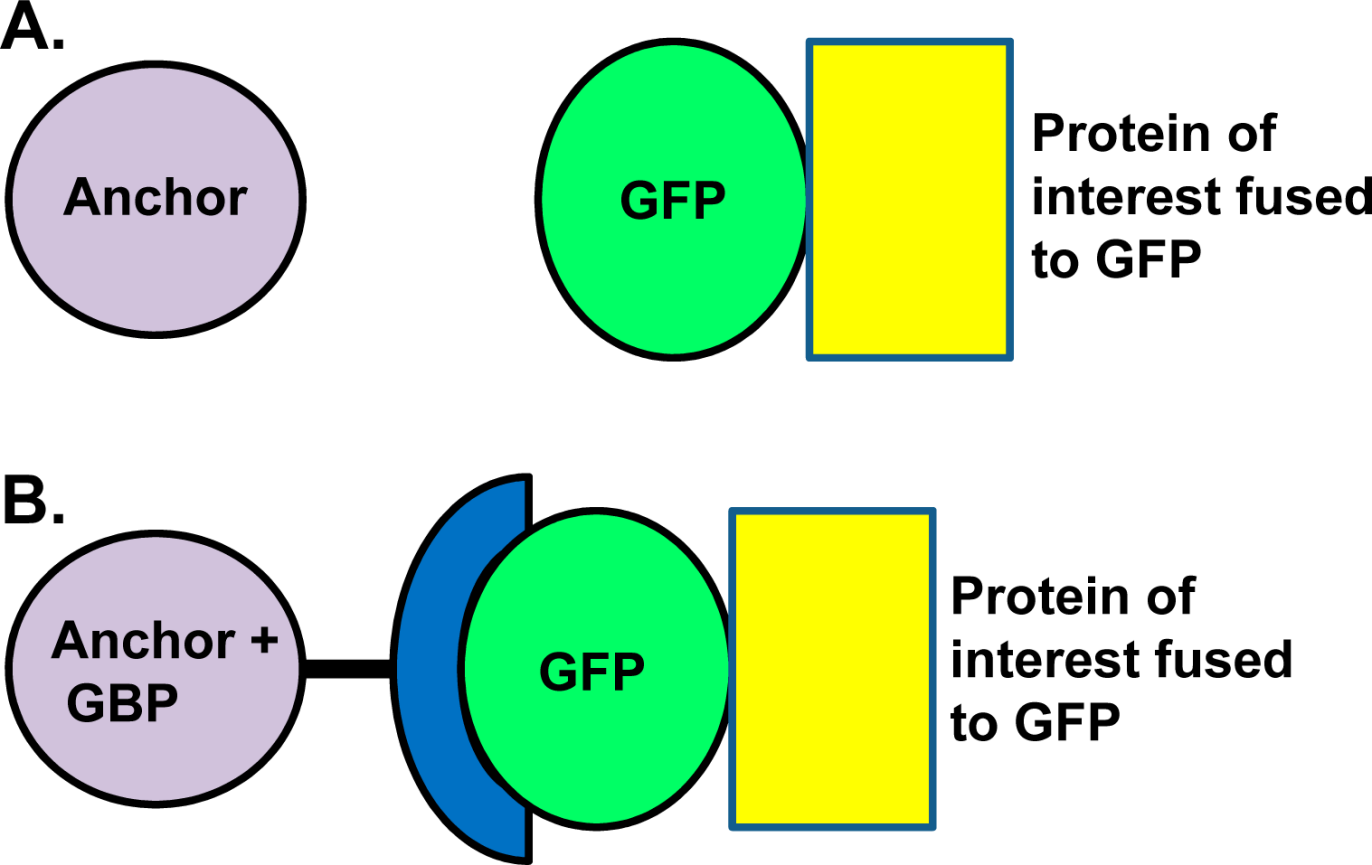
Protein retargeting strategy using the GBP-GFP system. (A) GFP-tagged protein of interest (yellow) to be retargeted to the anchor protein (purple). (B) Anchoring protein fused with GBP (blue) attracts GFP-tagged target protein via an interaction between GFP and GBP.

We have adapted the GFP-GBP system for protein retargeting in *A*. *nidulans*. In this system, the protein of interest and an anchoring protein are tagged with GFP or GBP (Fig 1). Because many labs have already endogenously GFP-tagged numerous proteins in *A*. *nidulans*, we chose to tag retargeting anchoring proteins with GBP. We used either the GBP sequence alone or along with mRFP (GBP-mRFP). The GBP-mRFP constructs enables visualization of the anchor protein in addition to the GFP-tagged protein that is to be retargeted. The GBP alone version allows a red-tagged experimental protein to also be utilized; when investigating if a GFP retargeted protein is able to attract a red-tagged interacting protein for example. We amplified the GBP or the GBP-mRFP sequence by PCR and added a 15-nucleotide sequence encoding the GA_5_ linker sequence at their 5’ ends. The GA_5_ linker between the anchor protein and GBP protein provides a flexible linker [9]. The final GBP plus nutritional marker gene cassettes were cloned into plasmid vectors (Table 2). To generate replacement constructs using fusion PCR [8], universal primers previously generated can be employed to amplify the GBP-tagging cassettes because of their universal 5’ and 3’ sequences [9, 11, 12]. Once generated, gene replacement DNA constructs are targeted via homologous recombination [15] so that GBP is expressed as a tag fused to the endogenous, and only copy, of the anchor protein. GBP tagged strains can be used in crosses to introduce the GBP anchoring protein into different GFP-tagged backgrounds or the GBP constructs can be directly transformed into strains already expressing GFP-tagged proteins.

### Retargeting of GFP proteins to septa using the SPA10-GBP anchor

**Fig 2:**
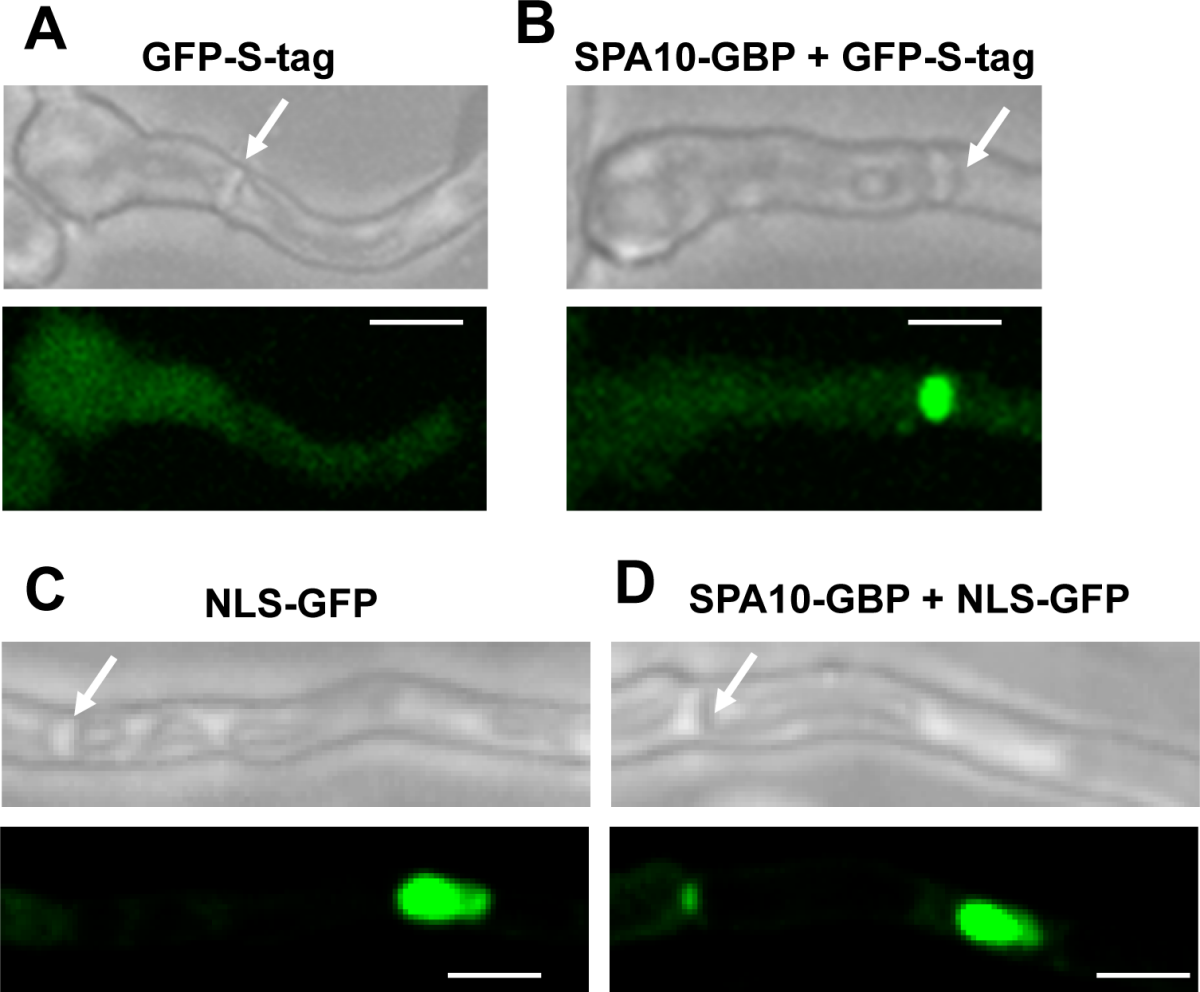
SPA10-GBP targets GFP-S-tag and NLS-GFP to septa. (A) Localization of GFP-S-tag in a WT cell as a uniform cytoplasmic signal with no localization at the septa. (B) Retargeting of GFP-S-tag to septa in the presence of SPA10-GBP anchor. (C) Localization of NLS-GFP in a WT cell with no localization at the septa. (D) Retargeting of NLS-GFP to septa in the presence of SPA10-GBP anchor. White arrows indicate septa. Scale bar, ~ 5μm.

We tested the ability of GBP-tagged proteins to retarget GFP-tagged proteins within *A*. *nidulans* cells. Because of the defined and distinctive location of septa within cells we first chose to try and retarget GFP-tagged proteins to septa. *A*. *nidulans* vegetative cells are syncytial and contain multiple nuclei in a common cytoplasm. Cells grow in a highly polarized manner from a single growing tip and, when first growing from spores, undergo 2–3 rounds of synchronous mitosis without undergoing septation [16–18]. The elongated syncytial cells then undergo septation after subsequent mitosis which generates cell compartments containing multiple nuclei separated by septa. Septation is incomplete in *A*. *nidulans* so that septa contain a central ~50 nm diameter pore which enables direct cell-cell communication [13]. Septa contain a set of Septal Pore Associated proteins termed SPA proteins [13], first identified in *Neurospora crassa* [19], many of which are intrinsically disordered proteins. Several SPA proteins have been identified in *A*. *nidulans* including SPA10 which locates to forming septa, remains stably associated at mature septa [13] and plays roles in nucleating microtubules from septa [20]. We therefore chose SPA10-GBP as a stable septal pore anchor for GFP-tagged proteins that do not normally associate with septa. Two GFP proteins were tested, cytoplasmic GFP-S-Tag [11] and nuclear NLS-GFP [13]. GFP-S-Tag is expressed from the endogenous AN1553 promoter at low levels throughout cells (Fig 2A) while NLS-GFP (Nuclear Localization Sequence-GFP) expressed from the *gpdA* promoter is transported into nuclei via its NLS (Fig 2C). Neither of these GFP chimeras normally locates to septa (Fig 2A and 2C). However, when expressed in SPA10-GBP cells, some GFP-S-Tag and NLS-GFP located to septa indicating successful retargeting of these GFP proteins to septa via SPA10-GBP (Fig 2B and 2D). Although some NLS-GFP was retargeted to septa, much of it was still present within nuclei (Fig 2D). The continued nuclear accumulation of NLS-GFP could be due to the high efficiency of nuclear transport such that most NLS-GFP is effectively imported into nuclei and not available for retargeting to septa. Also, the difference in abundance between SPA10 and NLS-GFP could limit the amount of NLS-GFP that can be retargeted as might the accessibility of the GBP moiety within the septal structure.

**Fig 3:**
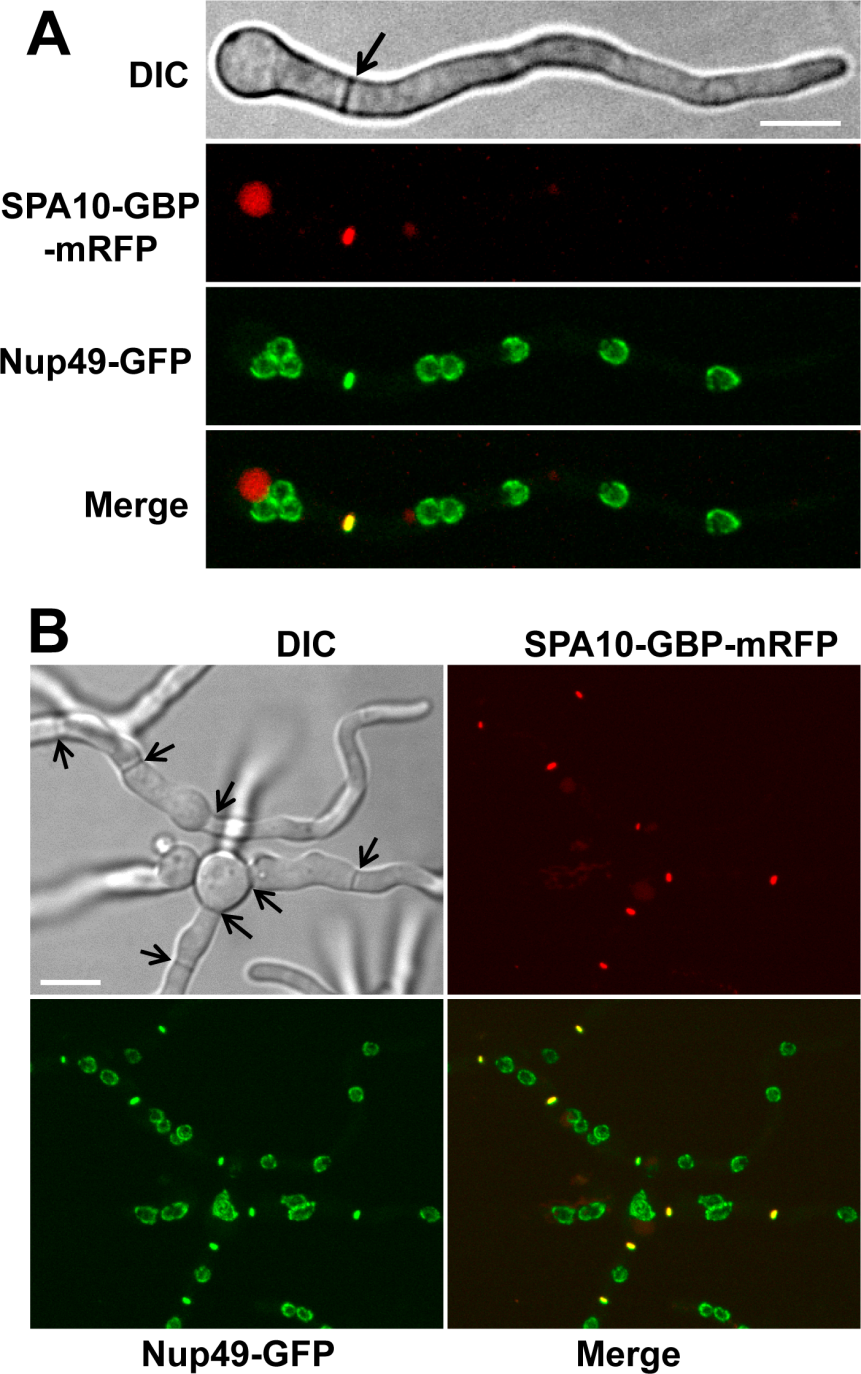
SPA10-GBP targets Nup49-GFP to mature septa. (A) Localization of Nup49-GFP to septa (arrow) in the presence of the SPA10-GBP-mRFP anchor as well as around the NPCs of the eight nuclei in the cell. Note that the round structure in the red channel (SPA10-GBP-mRFP) is a non-septal vacuolar signal. The vacuolar signal does not correspond with any Nup49-GFP signal and appears red in the merged image while the red and green signals co-localize at septa to appear yellow. (B) A larger group of SPA10-GBP-mRFP + Nup49-GFP cells with 8 septa present (visible septa in the DIC channel arrowed) all showing co-localization between SPA10-GBP-mRFP and Nup49-GFP signals. Scale bars, ~ 5μm.

**Fig 4:**
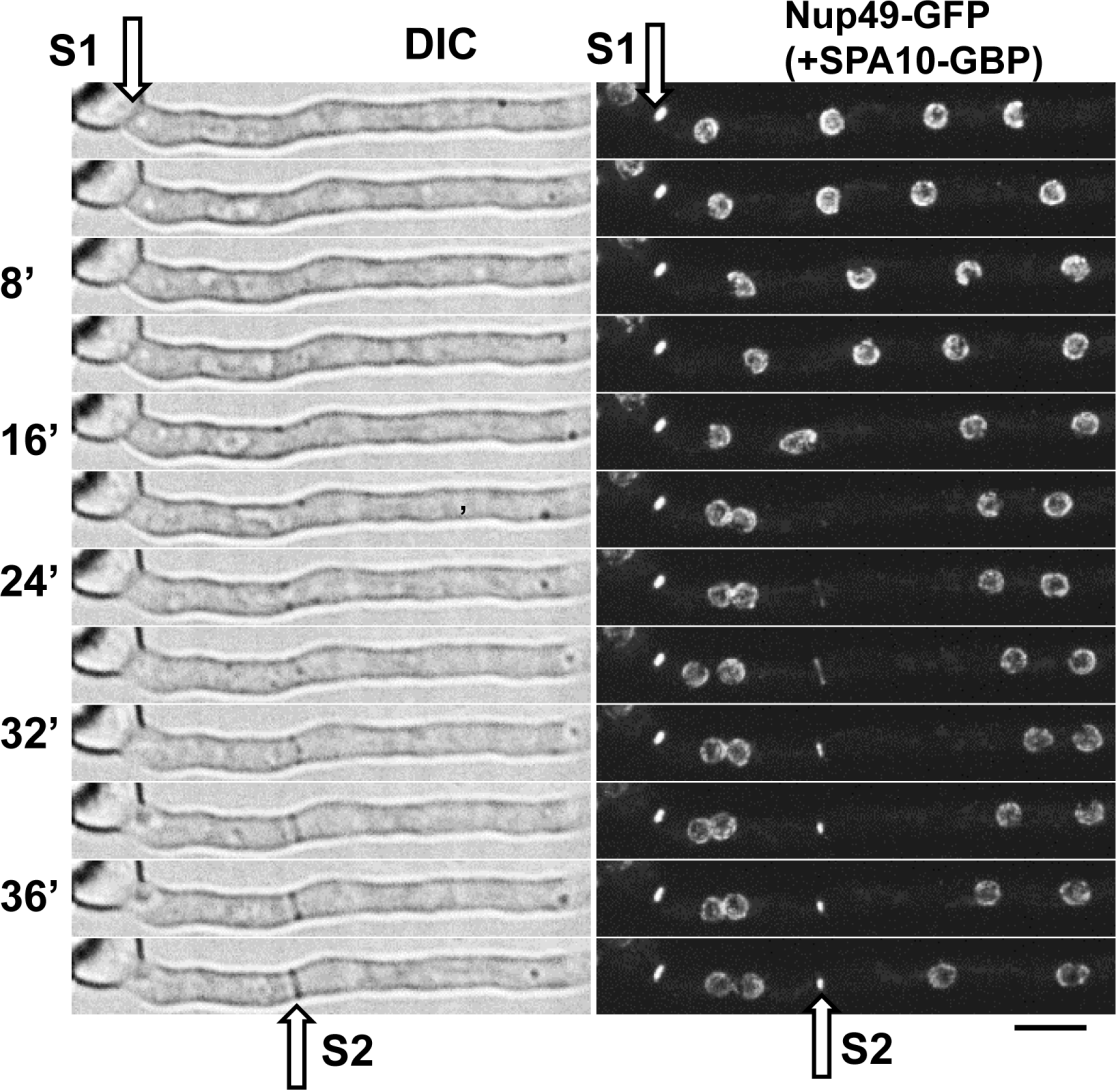
SPA10-GBP targets Nup49-GFP to forming septa. (A) Localization of Nup49-GFP at a mature septa (S1) and to a forming septa (S2) in the presence of the SPA10-GBP anchor as well as to the NPCs around nuclei in the cell. Scale bar, ~ 5μm.

We next addressed if an endogenously GFP-tagged protein of the nuclear pore complex (NPC) could be retargeted to septa via SPA10-GBP. In this case we utilized a GBP-mRFP cassette to generate the SPA10-GBP-mRFP gene replacement construct. SPA10-GBP-mRFP located at all mature septa (Fig 3A and 3B). For the GFP-tagged NPC protein we used Nup49, a non-essential peripheral component of NPCs [7]. Nup49-GFP locates exclusively at NPC structures which are embedded in the nuclear envelope. However, an additional location of some Nup49-GFP at septa was apparent in cells expressing SPA10-GBP (Fig 3). During live cell imaging, we observed Nup49-GFP also locating to forming septa in the same manner as previously shown for SPA10-GFP [13]. During septum formation, like SPA10 [13], Nup49-GFP locates to a closing ring structure (Fig 4), representing the contractile actomyosin ring (CAR) which is known to precede and drive septation which was also observed in the live cell DIC imaging (Fig 4, DIC and S1 Video). The data reveal that SPA10-GBP can retarget Nup49-GFP during the formation of new septa and that, like SPA10, Nup49-GFP remains at mature septa after their formation.

### Retargeting of Nup49-GFP to mitochondria using the TOM20-GBP anchor

We next asked if a higher abundance protein tagged with GBP at a non-septal location might retarget more Nup49-GFP away from its normal location at NPCs. GBP was therefore fused with Tom20, a transmembrane mitochondrial translocase protein of the outer mitochondrial membrane with a cytosol-exposed C-terminus [21]. Tagging Tom20 C-terminally with GBP-mRFP should provide GFP binding sites at the outer surface of mitochondria within the cytoplasm. Fungal mitochondria are tubular shaped organelles that extend throughout the length of cells [22]. Our initial Tom20 experiments utilized a strain expressing both Tom20-GBP-mRFP as well as NLS-DsRed to act as a marker for nuclei [23]. In a strain expressing Nup49-GFP and NLS-DsRed, the Nup49 signal was observed exclusively around the nuclear periphery with the nuclei containing NLS-DsRed (S1B Fig). In a Tom20-GBP-mRFP plus NLS-DsRed strain mitochondria extended throughout the cytoplasm as expected (S1A Fig). Most noticeably, in the strain expressing Tom20-GBP-mRFP plus NLS-dsRed and Nup49-GFP, little Nup49-GFP signal was apparent around nuclei. Instead Nup49-GFP was retargeted throughout the cytoplasm in association with the Tom20-GBP-mRFP mitochondrial signal (S1C Fig).

**Fig 5:**
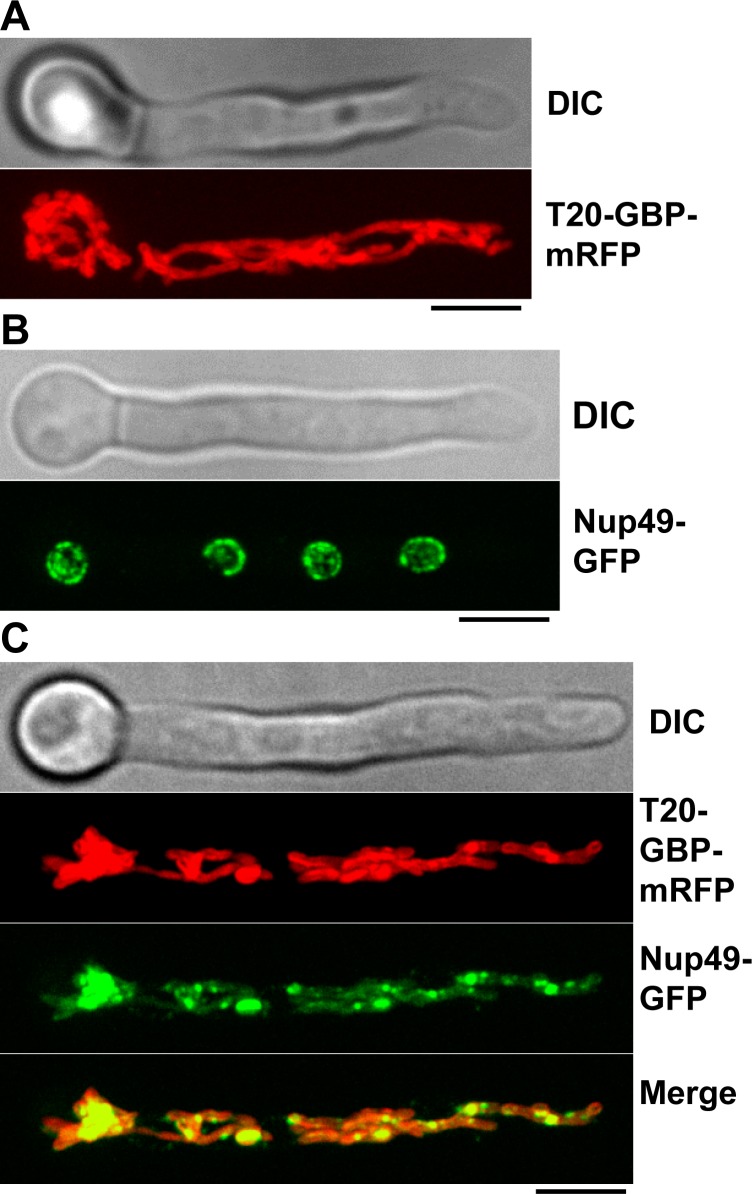
Retargeting the nuclear pore complex protein Nup49-GFP to mitochondria using Tom2-GBP-mRFP. (A) Tom20-GBP-mRFP (T20-GBP-mRFP) locates to tubular structures along the length of cells as is typical of mitochondrial markers in *A*. *nidulans*. (B) Localization of the NPC protein Nup49-GFP exclusively to NPCs at the nuclear envelope of nuclei during interphase. (C) Retargeting of Nup49-GFP away from the nuclear periphery to throughout the cytoplasm co-localizing with Tom20-GBP-mRFP. Scale bar ~5μM.

While the NLS-DsRed signal helps define the location of nuclei, because of its relative brightness, it also hinders somewhat the visualization of Tom20-GBP-mRFP (S1 Fig). We therefore generated strains with Nup49-GFP or Tom20-GBP-mRFP or with both Nup49-GFP plus Tom20-GBP-mRFP in the absence of NLS-dsRed. In the singly tagged strains, Tom20-GBP-mRFP was observed throughout cells in a tubular pattern typical of fungal mitochondria (Fig 5A) and Nup49-GFP located exclusively around nuclei (Fig 5B) as expected. A markedly different distribution of Nup49-GFP was observed in the double tagged Nup49-GFP plus Tom20-GBP-mRFP strain. Instead of concentrating solely around the nuclear periphery Nup49-GFP was relocated throughout the cytoplasm in association with the mitochondrial Tom20-GBP-mRFP signal (Fig 5C).

**Fig 6:**
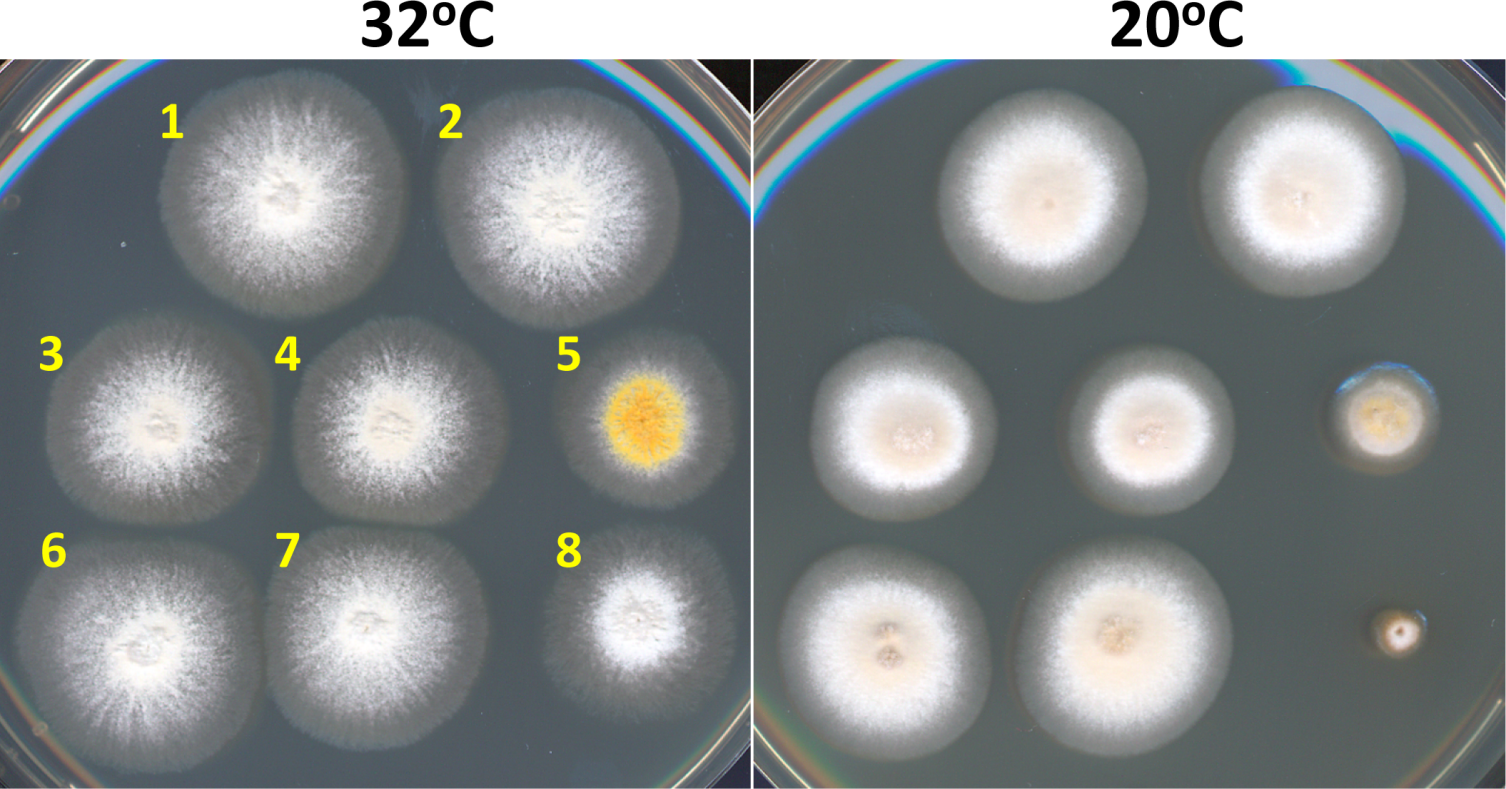
Retargeting of Nup49-GFP to mitochondria via Tom20-GBP phenocopies *Δnup49* cells causing cold-sensitivity. *A*. *nidulans* strains were replica point inoculated then incubated at 32°C for 3 days or at 20°C for 7 days. Strains are: 1. CDS1008 ΔKu. 2. CDS746 prototroph. 3. SGS365 Tom20-GBP-mRFP. 4. SGS367 Nup49-GFP. 5. SO759 Δ*nup49*. 6. SO1749 Spa10-GBP-mRFP + Nup49-GFP. 7. SGS134 SPA10-GBP-mRFP. 8. SGS374 Tom20-GBP-mRFP + Nup49-GFP. Note that strain SO759 (Δ*nup49*) carries a *yA* mutation (yellow spores) while all others carry a *wA* mutation (white spores). Full genotypes are given in Table 1.

The apparent near complete relocation of Nup49-GFP from NPCs to mitochondria might be expected to cause a cold sensitive growth phenotype as does the null allele [7]. We therefore tested control strains and those with different combinations of GBP and GFP tagged proteins for potential cold sensitivity (Fig 6). Notably the only strains tested to have a cold sensitive phenotype were the Δ*nup49* and Tom20-GBP-mRFP + Nup49-GFP strains (Fig 6).

## Conclusions

A series of GBP plasmid cassettes have been generated to enable generation of gene replacement GBP constructs via fusion PCR. The GBP gene replacement constructs generated can be used to endogenously tag chosen anchoring proteins with GBP to retarget GFP tagged proteins away from their normal cellular locations. It should also be noted that, depending on the relative strengths of the associations of the native anchor and target proteins, that the “anchor” protein-GBP may be retargeted to the target protein-GFP location. Using SPA10, a protein that locates to forming and mature septa, tagged with GBP (SPA10-GBP) we successfully targeted cytoplasmic GFP-S-Tag and nuclear GFP-NLS proteins to septa. Endogenously tagged Nup49-GFP was also retargeted to SPA10-GBP at both forming and mature septa. We further used Tom20, a component of the mitochondrial translocase of the outer membrane complex, tagged with GBP (Tom20-GBP) to successfully relocate the majority of Nup49-GFP away from NPCs to the surface of mitochondria causing marked cold sensitivity similar to the effects of the null Δ*nup49* mutation. These cassettes and GBP-tagged strains will enable further application of the GBP system to experimentally manipulate linkage of additional proteins in *A*. *nidulans* and other filamentous fungi.

## Methods

### General techniques

The techniques for classical genetics, strain construction, media preparation, culture and transformation of *A*. *nidulans* were carried out as described previously [7, 9, 24, 25]. Strains used in the study, genotypes and the method of generation are listed in Table 1.

### Generation of GBP cassette vectors

Plasmids encoding the GBP cassettes were produced as briefly described earlier [1]. The GBP encoding clone was obtained from Heinrich Leonhardt, Ludwig Maximilian University, Munich, Germany. The GBP (or GBP-mRFP) sequence was amplified from plasmid pc1336 using a forward primer that carries the GA_5_ linker sequence (GGAGCTGGTGCAGGCGCTGGAGCCGGTGCC) as a 5’ overhang. Genes encoding the nutritional marker genes *pyrG* or *pyroA* were amplified from *Aspergillus fumigatus* DNA to reduce the chance of integration of the construct into the native *A*. *nidulans pyrG* or *pyroA* loci [8, 15]. The final GBP cassettes were made using fusion PCR to fuse fragments encoding GA_5_-GBP or GA_5_-GBPmRFP and either the *pyrG* or *pyroA* genes as selection markers [8]. Fusion PCR was carried out using the PfuX enzyme compatible for cloning using the pCR-Blunt II-TOPO system (Invitrogen). The fusion PCR products were extracted from an agarose gel, purified and cloned into a pCR-Blunt II-TOPO vector (Invitrogen). The cloned products were transformed into chemically competent DH5α cells and selected with 50 μg/mL Kanamycin on Luria-Bertani plates. The resulting clones were tested for the presence of the insert by restriction digestion. The clones were further confirmed by sequencing on the Watson and Crick strands of the positive clones. The GBP with either *pyrG* or *pyroA* and the GBP-mRFP with either *pyrG* or *pyroA* base cassettes can now be amplified from different plasmids (Table 2) using the same universal primers HP116 (GGAGCTGGTGCAGGCGCTGGAGCC) and FN01 (CTGTCTGAGAGGAGGCACTGATGC) when generating GBP gene replacements constructs [9, 12].

### Generation of GBP-fused anchoring proteins

For generation of proteins fused with GBP, the GBP/marker cassettes were amplified from plasmids created above using the universal primers. Replacement constructs were generated using fusion PCR to fuse GBP to the 3’ end of SPA10 or Tom20 coding sequence before their stop codon. This results in the generation of a chimeric protein with GBP fused endogenously to the C-terminus of the anchoring proteins coding for SPA10-GBP or SPA10-GBP-mRFP and Tom20-GBP or Tom20-GBP-mRFP. Positive *A*. *nidulans* transformants expressing the GBP-fused anchoring proteins were generated after selection for either the *pyrG* or *pyroA* nutritional markers. Cells carrying GBP-fused proteins were confirmed by diagnostic PCR at the endogenous locus of the anchoring proteins [7].

### Imaging

Imaging of *A*. *nidulans* expressing fluorescently-tagged proteins was carried out using two different spinning disk confocal systems as described previously [1]. The growth conditions of *A*. *nidulans* cells for imaging have also been described earlier [26]. For imaging of septal pore-associated proteins, cells were allowed to undergo at least 2 rounds of mitosis following germination to allow for septum formation.

## Supporting information

S1 Fig Tom20-GBP retargets the nuclear pore complex protein Nup49-GFP to mitochondria. (A) Localization of Tom20-GBP-mRFP in relation to the nuclear marker NLS-dsRed. (B) Localization of the NPC protein Nup49-GFP in relation to nuclear NLS-dsRed. (C) Retargeting of Nup49-GFP from the nuclear periphery to the cytoplasm in cells expressing Tom20-GBP-mRFP with no apparent Nup49-GFP accumulation around the nuclear periphery. Scale bar, ~ 5μm.
(PDF)

S1 Video Live cell imaging shows some Nup49-GFP locating to forming septa via SPA10-GBP. Cells expressing both Nup49-GFP and SPA10-GBP were imaged every 2 minutes 23 times and the playback rate is 2 frames per second. Imaging was started just after completion of mitosis. The arrow marks the location of where the septum forms.
(AVI)
