# Supplementary material for: Tools for retargeting proteins within Aspergillus nidulans
Source: PLoS One. 2017 Dec 1;12(12):e0189077. doi: 10.1371/journal.pone.0189077 (PMC5711018; doi:10.1371/journal.pone.0189077)
Supplement: S1 Fig — (A) Localization of Tom20-GBP-mRFP in relation to the nuclear marker NLS-dsRed. (B) Localization of the NPC protein Nup49-GFP in relation to nuclear NLS-dsRed. (C) Retargeting of Nup49-GFP from the nuclear periphery to the cytoplasm in cells expressing Tom20-GBP-mRFP with no apparent Nup49-GFP accumulation around the nuclear periphery. Scale bar, ~ 5μm. (PDF) [file pone.0189077.s001.pdf]

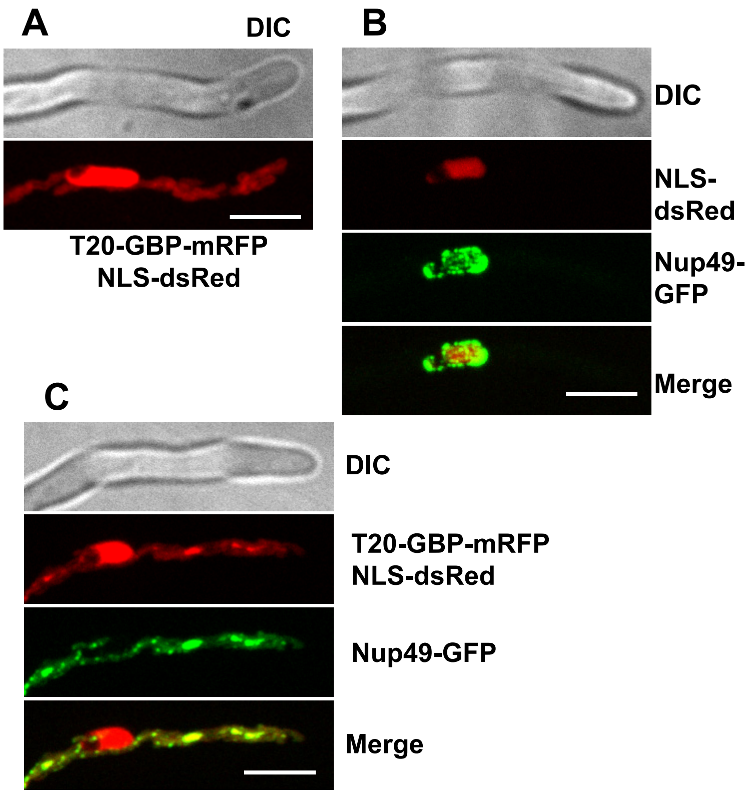

Supplementary Figure S1. Tom20-GBP retargets the Nuclear Pore Complex protein Nup49-GFP to mitochondria. (A) Localization of Tom20-GBP-mRFP in relation to the nuclear marker NLS-dsRed. (B) Localization of the NPC protein Nup49-GFP in relation to nuclear NLS-dsRed. (C) Retargeting of Nup49-GFP from the nuclear periphery to the cytoplasm in cells expressing Tom20-GBP-mRFP with no apparent Nup49-GFP accumulation around the nuclear periphery. Scale bar, ~ 5μm.
